# Sepsis Endotypes Defined By Cytokine Trajectory Analysis

**DOI:** 10.1186/2197-425X-3-S1-A46

**Published:** 2015-10-01

**Authors:** L Zhu, F Pike, LA Zhang, RS Parker, ED Mochan-Keef, D Swigon, G Clermont, the ProCESS Investigators, University of Pittsburgh

**Affiliations:** University of Pittsburgh, Graduate School of Public Health, Pittsburgh, United States; CRISMA Laboratory, Critical Care Medicine, Pittsburgh, United States; University of Pittsburgh, Critical Care Medicine, Pittsburgh, United States; University of Pittsburgh, Chemical Engineering, Pittsburgh, United States; University of Pittsburgh, Mathematics, Pittsburgh, United States

## Introduction

Several studies have linked cytokine levels to severity of sepsis and outcome, but the relative timing of biomarker levels, the septic process and outcomes is often unclear.

## Objectives

To determine whether there exists endotypes of sepsis defined by biomarker trajectories, and whether types of trajectories are associated with 60-day mortality.

## Methods

The Protocol-based Care in Early Septic Shock (ProCESS) trial prospectively enrolled 1341 patients with sepsis having a 19.3% 60-day mortality (Yealy et al., 2014). Biomarker levels were obtained soon after enrollment (t = 0) and at 6, 24 and 72 hours in a convenience cohort of 638 patients having 17.1% mortality. We used trajectory analysis to cluster IL-6, IL-10, and joined IL-6 and IL-10 trajectories in the 638 patients having at least one measurement of IL-6 or IL-10, in patients with markers rising between 0 and 6 hours (presumably earlier in the course of sepsis), and in patients with monotonically decreasing biomarker levels (presumably later in the course of sepsis). Trajectories were described as increasing from t = 0 to 6 hours, decreasing or flat. 60-day mortality was compared between clusters to determine the association between cytokine trajectory and mortality.

## Results

600 patients had measurements at 0 and 6 hours, 570 had at least 3 measurements, but only 170 patients had measurements at all time-points. There was no association between stated times of first symptoms (average 4.0 days), times between emergency department arrival and enrolment (average 3.1 hours) and whether trajectories were increasing or decreasing at the first two time points. Comparing trajectory types of the first two time points (t = 0 and 6), patients with rising profiles of both IL-6 and IL-10 had the highest mortality (30.8%, Table), followed by patients with either rising IL-10 (25.8%) or rising IL-6 (27.9%). in trajectory analysis of all four time-points, the cluster of patients with elevated levels of both cytokines and blunted decreases had the highest mortality (>50%). Patients with high cytokine level at enrollment but rapidly decreasing levels of IL-6 had a favorable outcome. Flat and persistently low trajectories were associated with the lowest mortality. There exists a significant cohort of patients with a minimal cytokine response that died, and a smaller cohort of patients with vigorous and sustained response, that survived.

**Table 1:** Table 1

	IL-6 increasing 191 (23.6%)	IL-6 decreasing 359 (13.9%)	IL-6 flat 50 (6.0%)
IL-10 increasing 147 (29.3%)	78 (30.8%)	68 (27.9%)	1 (0%)
IL-10 decreasing 297 (15.5%)	62 (25.8%)	222 (12.6%)	13 (15.6%)
IL-10 flat 155 (5.8%)	51 (9.8%)	69 (4.3%)	35 (2.9%)

*[T = 0 to 6 hours trends: N of patients (mortality%)]*

## Conclusions

In this exploratory analysis, patients with rising levels of cytokines, or persistently elevated levels of cytokines at t≤6 hours are at high risk of mortality. Endotypes of patients with unexpected outcomes, non-survivors with blunted cytokine response, or survivors with exuberant response, deserve more detailed study.

## Grant Acknowledgment

NIH R01-GM105728
